# Enrichment of the fetal fraction in non-invasive prenatal screening reduces maternal background interference

**DOI:** 10.1038/s41598-018-35738-0

**Published:** 2018-12-05

**Authors:** Bo Liang, Hong Li, Quanze He, Haibo Li, Lingyin Kong, Liming Xuan, Yingying Xia, Jingjing Shen, Yan Mao, Yixue Li, Ting Wang, Yi-Lei Zhao

**Affiliations:** 1 State Key Laboratory of Microbial Metabolism, Joint International Research Laboratory of Metabolic and Developmental Sciences, MOE-LSB and MOE-LSC, Department of Bioinformatics and Biostatistics, School of Life Sciences and Biotechnology, Shanghai Jiao Tong University, 800 Dongchuan Road, Shanghai, 200240 China; 2 Center for Reproduction and Genetics, The Affiliated Suzhou Hospital of Nanjing Medical University, 26 Daoqian Street, Suzhou, Jiangsu 215002 China

## Abstract

Measurement of cell-free fetal DNA (cffDNA) is an indispensable process for non-invasive prenatal screening (NIPS). According to recent studies, cffDNA in maternal plasma can be enriched for various lengths of fragments, and a sufficient amount of cffDNA can effectively eliminate background interference on the part of maternal DNA. Therefore, we developed a simple and effective separation method, improved NIPS (iNIPS), that enriches the fetal fraction and improves the accuracy of NIPS for fetal aneuploid detection. We adopted a novel strategy to achieve enrichment of 125–135 bp cell-free DNA (cfDNA) by e-gel electrophoresis. To evaluate clinical performance, we compared NIPS and iNIPS results from 2153 retrospective clinical samples. Of the 22 samples with NIPS results of “no call”, 17 samples were reclassified as “unaffected” (9 cases of chr13, 5 cases of chr18, and 3 cases of chr21); 2 samples remained classified as “no call” (1 case of chr18 and 1 case of chr21); and 3 samples were identified as T21 by iNIPS. The average increase in abundance of cfDNA fragments of 125–135 bp was 2.5 times, and the average decrease in maternal background interference was 1.3 times. On this basis, the detection of fetal aneuploidy was highly improved with the fetal fraction as low as 2%; iNIPS achieved 100% sensitivity and 99.90% specificity in retrospective samples.

## Introduction

The discovery of cell-free fetal DNA (cffDNA) in maternal plasma has greatly promoted the development of non-invasive prenatal screening (NIPS) applications^1^, including chromosomal microdeletion detection, microduplication detection^2–5^, aneuploidy detection^6–9^ and monogenic disease^2,10–13^. The concentration of cffDNA is critical for the accuracy of these tests^2,12,13^. Nevertheless, the concentration of cffDNA in maternal plasma is very low, accounting for only 2–20% of the total maternal plasma cell-free DNA (cfDNA)^10,11,14^, with individual differences. Furthermore, cffDNA is mixed with maternal-derived cfDNA that produces significant background interference. These limitations restrict the application of cffDNA. Current NGS methods for NIPS require that the proportion of cffDNA fragments in the total free plasma DNA fragments of pregnant women be greater than 4%^13^. However, in approximately 1–3% samples, the fetal fraction is less than 4%^13^. In these samples, the positive sample detection rate is lower than 62.10%^15^. Although deeper sequencing can improve the accuracy of low fetal fraction samples, such methods are more expensive^2,13^. Furthermore, placental mosaicism may cause inconsistencies in aneuploidy detection, leading to false positives by significantly altering the z-score of the involved chromosomes. Therefore, for more accurate detection results, it is necessary to eliminate the background interference of maternal-derived cfDNA by increasing the abundance of cffDNA.

Recent studies have shown that when lengths of cffDNA fragments are less than 300 bp, approximately 50% of the cffDNA fragments are located within the range of 100–300 bp, and approximately 20% of maternal cfDNA fragments are greater than 300 bp^16–18^. Therefore, cffDNA can be enriched by collecting maternal plasma cfDNA fragments with lengths of less than 300 bp. Two previous studies used gel electrophoresis-based size separation to filter out the fragments larger than 300 bp to achieve fetal concentration enrichment^19,20^. Another study found that the size distribution of fetal and maternal cfDNA had several peaks, including 166 bp, 143 bp and 10 bp at the minimum interval. The significant difference between fetal and maternal cfDNA was that the main maternal peak was 166 bp and the main fetal peak was less than 150 bp^12^. Therefore, instead of removing large DNA fragments greater than 300 bp, we developed a novel strategy to improve the relative abundance of fetal-derived cfDNA by using e-gel electrophoresis to select fragments with a range less than 150 bp. The method is sufficient to produce large amounts of cffDNA to meet the requirements of routine NIPS. Our aim was to enrich the fraction of fetal DNA in the sequencing library and reduce false-negative and false-positive rates without increasing the cost of detection.

## Results

### Relationship between cfNDA fragment size and fetal fraction

**Figure 1:**
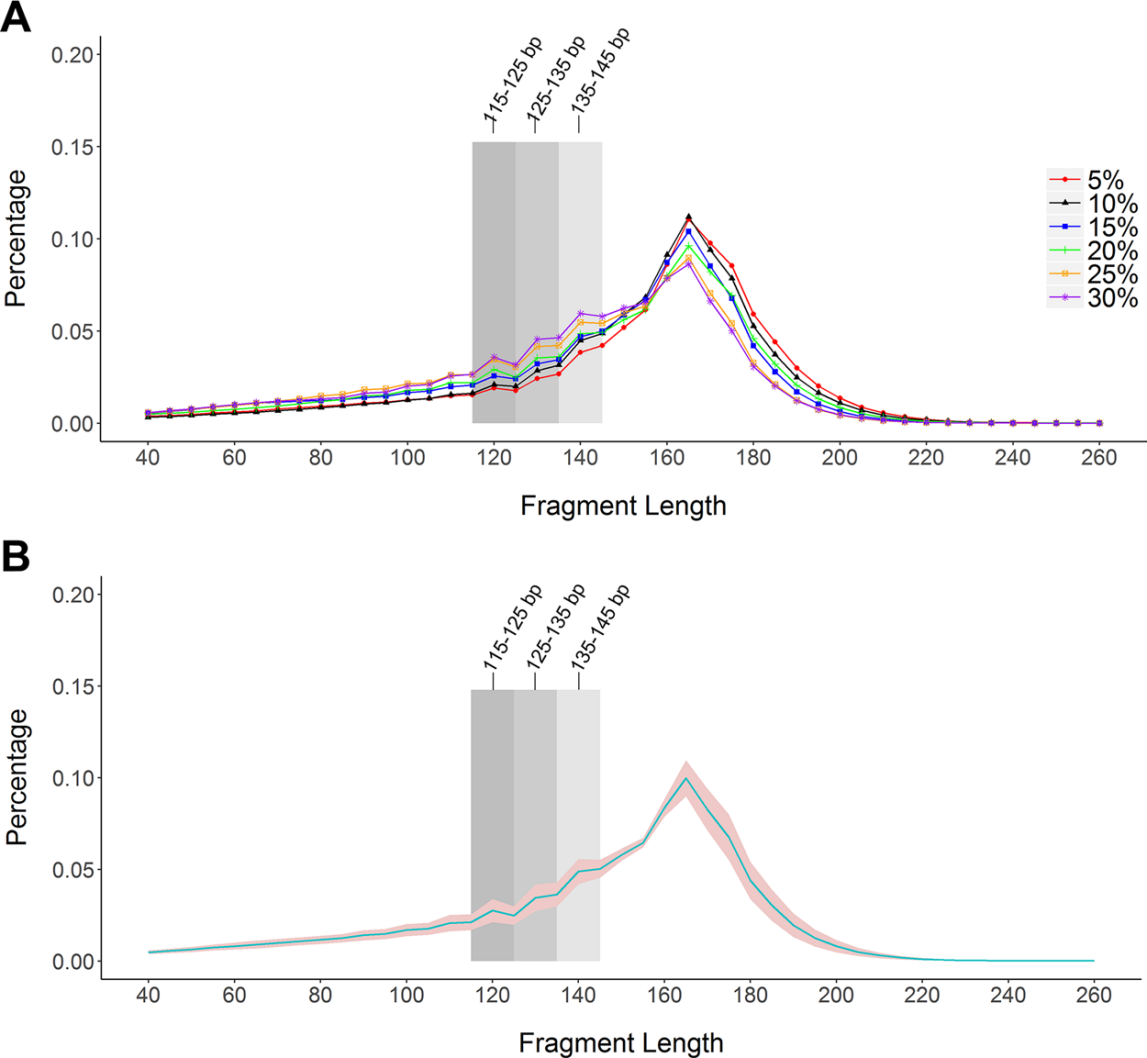
The cfDNA fragments lengths distribution of various fetal fractions. (**A**) Representative examples from maternal plasma with various fetal DNA fractions. (**B**) Aggregate of all samples. The blue line represents the mean read ratio of all samples, and the red region represents ± 1 SD.

**Figure 2:**
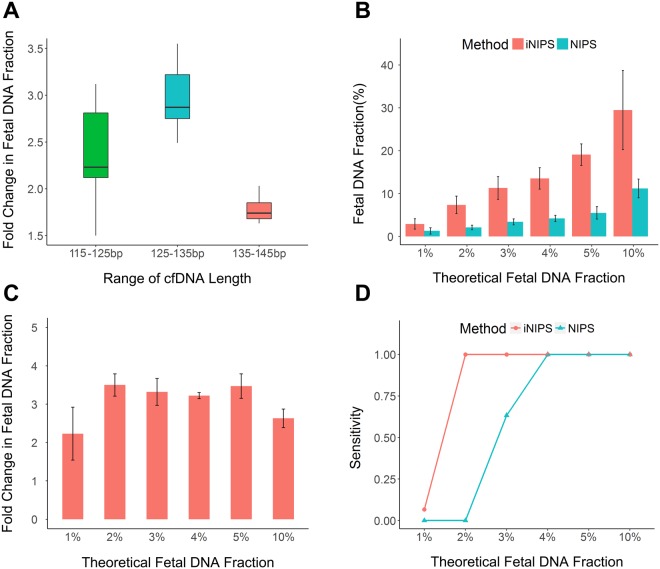
Fold increases in the fetal DNA fractions with various fragment sizes by iNIPS. (**A**) Increased fetal fractions by iNIPS for different sizes of cfDNA fragments. (**B**) Comparison of the fetal DNA fraction of NIPS and iNIPS. (**C**) Fold-change in fetal DNA fraction of NIPS vs iNIPS. (**D**) Comparison of sensitivities of NIPS and iNIPS for detecting abnormalities in low fetal DNA fractions.

The significant difference between fetal DNA and maternal DNA in cfDNA is that the fetal DNA has a reduced peak at 166 bp and an enhanced peak at less than 150 bp^2,12,21^. To determine the optimal size range of iNIPS, we compared the samples of various fetal fractions and found that the trend of three peak areas (115–125, 125–135 and 135–145 bp) was proportional to the fetal fraction (Fig. 1). Accordingly, we selected three ranges of DNA fragment sizes to enrich fetal DNA. We used the plasma of 9 pregnant women with male foetuses to calculate fetal fraction by the iNIPS method and compared the fetal DNA fragments in three size ranges. When the sizes of DNA fragments were in the ranges of 115–125, 125–135 and 135–145 bp, the median value of the fetal DNA fractions increased 2.23 times, 2.87 times and 1.74 times, respectively (Fig. 2A). Notably, fetal fraction was most abundant in the 125–135 bp fragment, indicating that cffDNA was mainly distributed between 125 and 135 bp. Therefore, in this study, we enriched DNA fragments with a size range of 125–135 bp.

### Validation of different fetal DNA fractions

In previous studies, approximately 1–3% of samples of the fetal fraction were found to be less than 4%, which was the most common cause of false-negative results^13^. For these samples, the detection rate for positive samples was less than 62.10%^15^. To determine the lower limit of fetal fraction of iNIPS, we verified the sensitivity and false-negative rate by analysing various T21 fetal DNA fractions, as shown in Fig. 2 (1%, 2%, 3%, 4%, 5% and 10%). The fetal DNA fraction in plasma cfDNA increased 2.23–3.50 times with a median value of 3.27 times, and the maternal DNA fraction in plasma cfDNA decreased 1.02–1.26 times with a median value of 1.10 times when using the iNIPS method (Fig. 2B,C). In this way, iNIPS accurately detected fetal aneuploidy in low fetal DNA fractions as low as 2%, with a sensitivity of 100% (Fig. 2D). In contrast, the routine NIPS method did not provide accurate results for detecting fetal DNA fractions less than 4%. This result implies that iNIPS could be a considerable supplementary method for NIPS in low fetal DNA fractions.

### Pre-clinical validation of iNIPS

**Figure 3:**
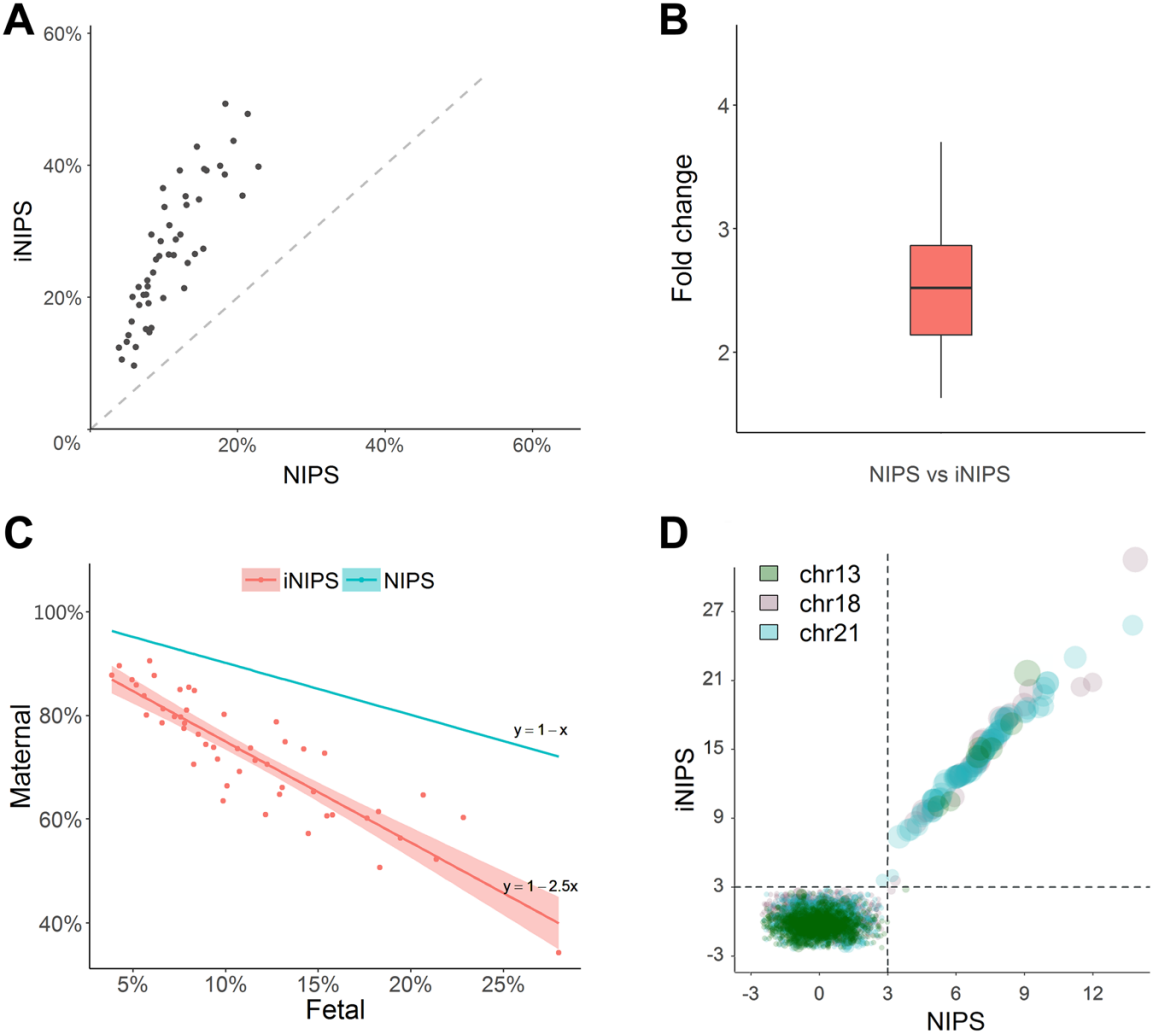
The efficiency of the iNIPS method. (**A**) Fetal DNA fraction comparison of the two methods for non-invasive genetic screening. (**B**) Fold increase in fetal DNA fraction using iNIPS. (**C**) The change in maternal DNA fraction. The blue line represents the NIPS maternal DNA fraction; red lines represents the iNIPS maternal DNA fraction that the fetal concentration enriched a mean value of 2.5 times. (**D**) NIPS Z-score VS iNIPS Z-score. The x-axis is the NIPS Z-score, and y-axis is the iNIPS Z-score. The circle size represents the ratio which was iNIPS Z-score divided by the NIPS Z-score.

Fifty male fetal libraries and 2 pregnant women libraries with chromosomal abnormalities were constructed, and 125–135 bp fragments were selected using E-Gel EX 2% gel. The selected size fragments were sequenced, and the fetal DNA fractions were analysed (Supplemental Table 1). Analysis of 50 male fetal libraries revealed that fetal DNA fraction increased 1.6–3.7 times (p-value < 2E-13), with a median value of 2.5 times (Fig. 3A,B), and the maternal DNA fraction decreased 1.04–2.10 times (p-value < 2E-16), with a median value of 1.3 times (Fig. 3C). Since plasma DNA contains both maternal DNA and fetal DNA, abnormalities in maternal chromosomes could lead to false-positive tests for fetal chromosomal abnormalities. The results of 2 cases of pregnant women with aneuploidy showed that, by iNIPS, the maternal chromosome abnormality fraction was decreased 1.2 times and 1.6 times (Supplemental Table 2). Therefore, iNIPS significantly decreased the z-score abnormality and reduced the false-positive rate caused by maternal aneuploidy DNA (Supplemental Table 2). In other cases, some euploid pregnant women have microreplications or microdeletions on their chromosomes. These alterations may interfere with fetal aneuploidy detection when maternal microduplication or microdeletion is greater than the threshold for diagnosing the fetal DNA fraction (Supplemental Table 3). With the enrichment of the fetal DNA fraction and the influence of maternal DNA decreased, the threshold for diagnosing the fetal DNA fraction was increased, and the maternal background interference was reduced.

### Clinical validation of iNIPS

**Figure 4:**
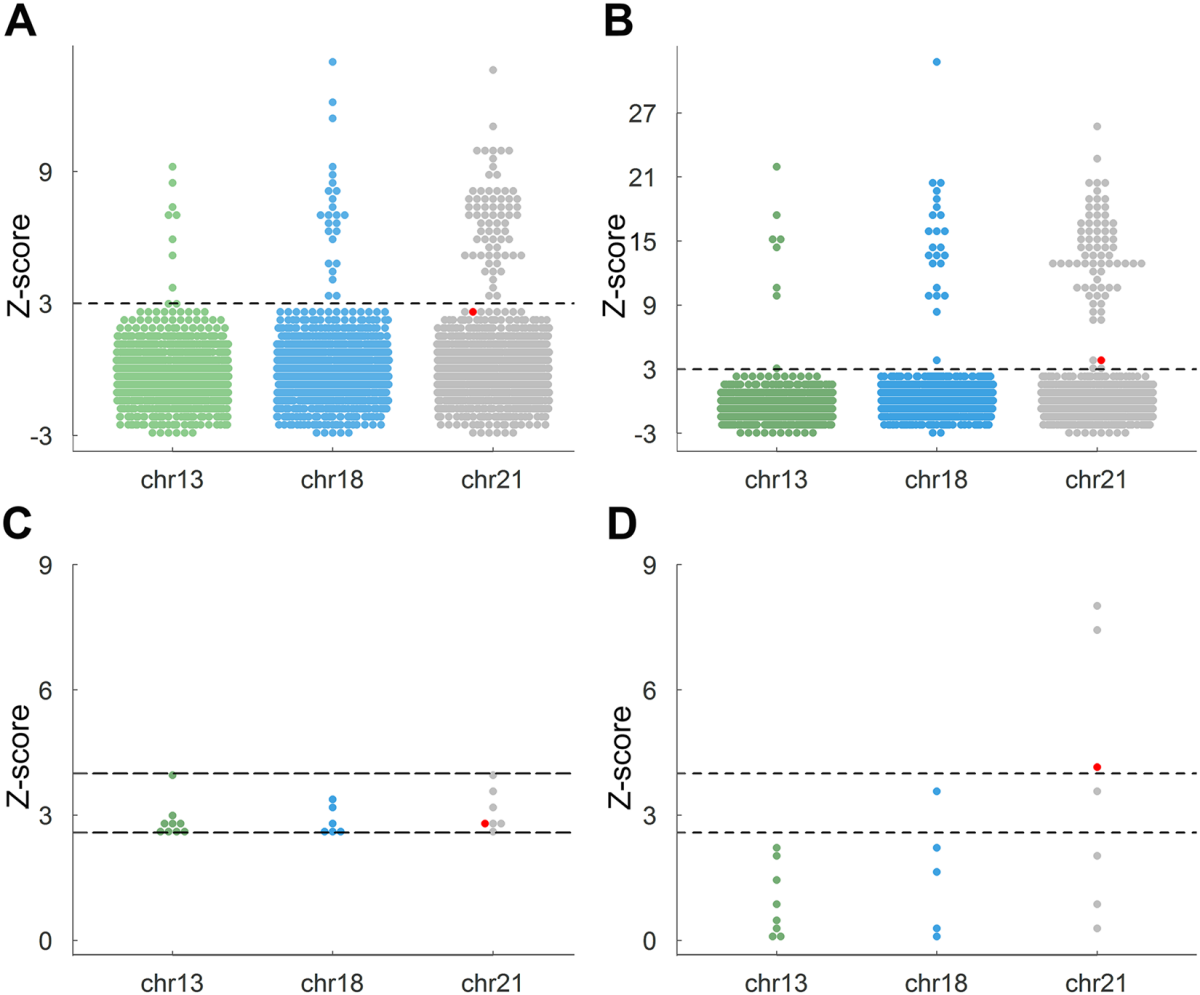
Z-score comparison by using NIPS and iNIPS. (**A**) NIPS Z-score. (**B**) iNIPS Z-score. The black dotted line means that z-score was 3.00. (**C**) The NIPS results showed that 22 samples were classified as “no call”. (**D**) Using iNIPS to analyse 22 “no call” samples. The red dot: one control that NIPS identified as “no call” was identified as T21 by iNIPS. The black dotted line means that the z-score was between 2.58 and 4.00.

We retrospectively selected 2153 clinical cases for sensitivity and specificity evaluation of iNIPS, of which 2023 were normal cases (healthy infants) and 130 were fetal aneuploid cases verified by karyotyping. As shown in Fig. 3D, all cases were evaluated using NIPS and iNIPS; the trend of Z-score was proportional to the fetal fraction in the aneuploid samples (T21/T18/T13). As shown in Fig. 4, the NIPS results showed that 22 samples were classified as “no call” (9 cases of chr13, 6 cases of chr18, and 7 cases of chr21), and their fetal fractions are listed in Supplement Table 4. Using iNIPS to analyse these samples, 17 samples were reclassified as “unaffected” (9 cases of chr13, 5 cases of chr18, and 3 cases of chr21); 2 samples were still classified as “no call” (1 case of chr18 and 1 case of chr21); and 3 samples were identified as T21. One control identified by NIPS as “no call” (z-score = 2.7 and Fetal Fraction = 16%) was identified by iNIPS as T21, in which the fetal DNA fraction was calculated using the Y chromosome percentage (z-score = 4.2 and Fetal Fraction = 44%), and the percentage of fetal T21 DNA was 5.6%, calculated using the high risk z-score^22^. According to the karyotype of amniotic fluid, we concluded that the false-negative sample was placental mosaicism (12.7% degree of mosaicism, 5.6% divided by 44%). Moreover, of the 2153 retrospective clinical cases, 1004 samples were from male fetuses, and their fetal cfDNA fractions validated by the fraction of Y chromosome ranged from 5.83 to 79.31 (median: 31.43) by iNIPS, compared with 3.33 to 55.49 (median: 14.58) by NIPS. Clinical results showed that iNIPS was more accurate than NIPS (Table 1). We used iNIPS to further validate “no call” samples classified by the routine NIPS method. In this retrospective study, the sensitivity and specificity of iNIPS for both euploidy and aneuploidy samples were 100% and 99.90%, respectively.

**Table 1:** Clinical results of the two methods.

Karyotyping (Quantity/case)	NIPS		iNIPS	
	Sensitivity*	Specificity*	Sensitivity*	Specificity*
T13 (7)	100% (7/7)	99.95% (2022/2023)	100% (7/7)	100% (2023/2023)
T18 (23)	100% (23/23)	99.90% (2021/2023)	100% (23/23)	99.95% (2022/2023)
T21 (70)	98.57% (69/70)	99.95% (2022/2023)	100% (70/70)	99.95% (2022/2023)
Other aneuploidy (30)	100% (30/30)	100% (2023/2023)	100% (30/30)	100% (2023/2023)
All (2153)	99.23% (129/130)	99.80% (2019/2023)	100% (130/130)	99.90% (2021/2023)

*The confidence interval is 99%.

## Discussion

The abundance of fetal DNA in maternal plasma is a crucial factor affecting the accuracy of maternal plasma DNA prenatal testing^2,12,13^. A study by Canick *et al*. suggested that the most common factor associated with false-negative results was a low fetal DNA fraction^13^. Because fetal DNA is generally shorter than maternal DNA in maternal plasma, many techniques have been developed to enrich the fetal DNA fraction by filtering out large DNA fragments^13^. These techniques include traditional gel electrophoresis^16–18^, combinations of PCR amplification with varied lengths of amplicons^19,20^ and microchip separation^23^. In two previous studies, fetal DNA was enriched by filtering out fragments larger than 300 bp by gel electrophoresis-based size separation before quantitative PCR^19,20^. Furthermore, adding beads to the sample to remove large DNA fragments during the construction of library was also an effective method to improve the reliability of low cfDNA samples^24^.

Instead of removing large DNA fragments, we developed a novel strategy to improve the relative abundance of fetal-derived cfDNA by selecting a certain range of fragments before NGS library construction. The size range of fragments had a major impact on the efficiency of enrichment of fetal DNA. PCR was used to amplify cfDNA, and e-gel electrophoresis was used to screen out the size of different cfDNA fragments. We found that the optimal size of DNA fragments was 125-135 bp, and these fragments were significantly enriched. We accurately detected chromosomal aneuploidies and reduced the detection requirement for the fetal fraction from 4% to 2%. Therefore, iNIPS can be used to further verify the “no call” sample in NIPS detection without increasing the cost of detection. In this study, we used 2153 retrospective clinical cases to determine whether iNIPS could be used in a clinical testing. We found that iNIPS had 100% sensitivity and 99.90% specificity for retrospective samples, reducing the “no call” samples from 22 to 2. Moreover, iNIPS improved the possibility of the detection of fetal chromosome abnormality in samples with confined placental mosaicism.

We developed the iNIPS method based on size selection of DNA fragments that increased the abundance of fetal DNA in maternal plasma and reduced the false-negative and/or false-positive rates. Nevertheless, size fractionation of E-gel electrophoresis is a time-consuming and labour-intensive process, increasing the complexity and instability of the method. More large-scale studies should be carried out to verify the applicability of this method in clinical application. Furthermore, the NIPS method remains insufficient to detect prenatal chromosomal microdeletions and microduplications because of low fetal DNA fractions in maternal plasma. As suggested by our study, increasing the amount of fetal DNA fraction in maternal plasma may improve the accuracy of iNIPS in detecting prenatal chromosomal microdeletions and microduplications.

## Conclusion

In this study, we used differences in the size of maternal and fetal DNA fragments in plasma to establish the size selection library for NGS. Although e-gel electrophoresis requires more time and manpower than routine NIPS operations, a precise size fractionation method can effectively improve the fetal fraction for prenatal screening. Compared with the NIPS method, the iNIPS method detects fetal chromosome aneuploidy more accurately and reduces maternal background interference. More importantly, with this method, we can further verify the “no call” sample detected in routine NIPS without increasing the cost of detection. In the future, we believe that iNIPS can expand the applications of NIPS in detecting microdeletions/microduplications and monogenetic diseases and that it may also play a role in areas other than prenatal screening, such as the prognosis of tumor therapy.

## Methods

### Ethics statement

This study was approved by the Reproductive Medicine Ethics Committee of Suzhou Municipal Hospital (approval No. K901001), and all studies were performed in accordance with relevant guidelines and regulations. Informed consent was obtained from all participants.

### Samples

We collected whole blood from 59 pregnant women (ages 20–37 years) with male foetuses (gestational ages 12–26 weeks) and 2 pregnant women with maternal chromosome abnormality from the Centre for Reproduction and Genetics of Suzhou Municipal Hospital for the NIPS analysis. Another 2153 retrospective clinical cases (ages 20–45 years, gestational ages 12–28 weeks, covering the first through third trimesters) with known fetal karyotypes or follow-up records were also examined to verify the precise size fractionation method, which we called improved-NIPS (iNIPS).

### cfDNA extraction and library construction

We extracted plasma from 10 ml whole blood samples of pregnant women using a two-step centrifugation process: tubes of blood were centrifuged at 1,600 × *g* for 10 min at 4 °C, and the plasma was then transferred to microcentrifuge tubes and centrifuged at 16,000 × *g* for 10 min to remove residual cells and obtain cell-free plasma. The cell-free plasma was stored at −80 °C before DNA extraction. DNA fragments were extracted from 0.6 ml cell-free plasma using the Circulating Nucleic Acid Kit (Qiagen, Germany). An Ion Plus Fragment Library Kit (Life Technologies, USA) for the Ion Proton platform was used to construct the sequencing library for each plasma sample, and the libraries were quantified on a Qubit Fluorometer.

### Library size selection

**Figure 5:**
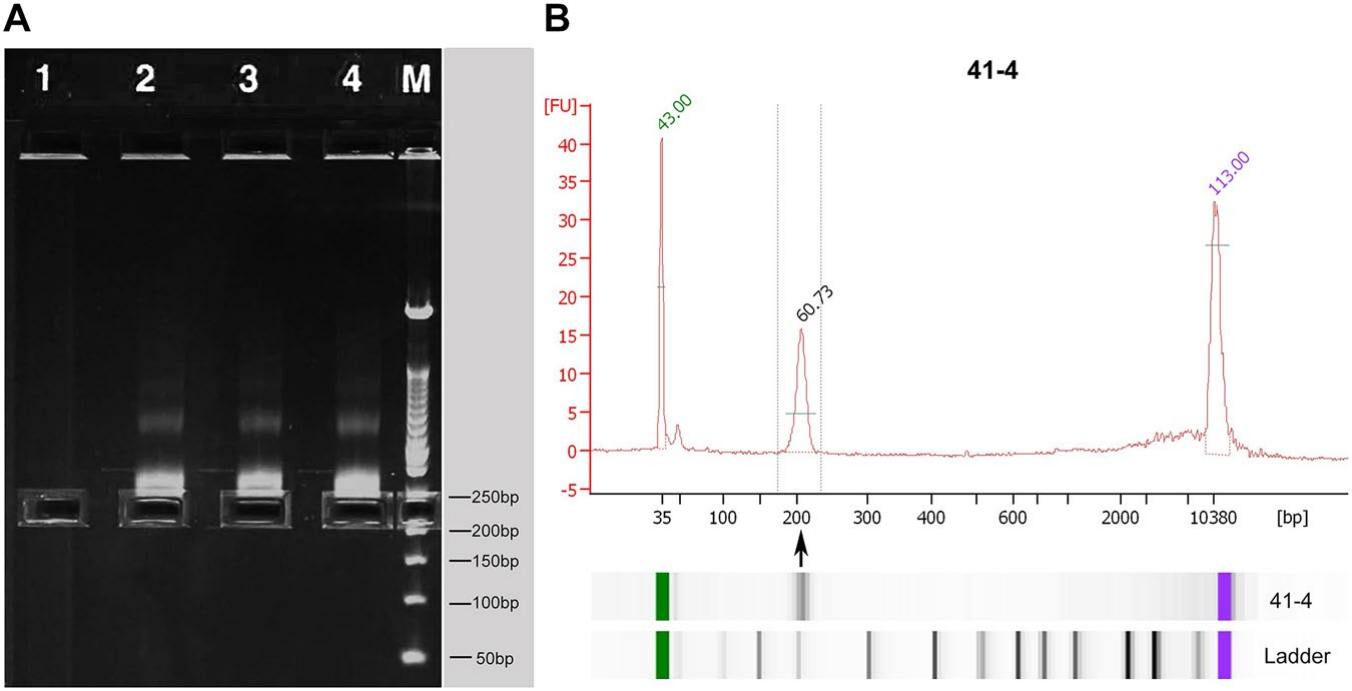
Selection of the library using E-gels and testing by Agilent 2100 Bioanalyzer. (**A**) Selection of the library between 190 and 240 bp (insert DNA was from 100 to 150 bp). (**B**) The selected library was tested using an Agilent 2100 Bioanalyzer.

We constructed a library containing 300–390 ng DNA for each sample. Libraries with barcodes were size-selected from within the range of 190–240 bp (insert DNA ranging from 100 to 150 bp) using E-Gel CloneWell Agarose Gels (Invitrogen, Carlsbad, CA, USA). A piece of E-Gels contains six effective wells, each well can run a mixed sample which contains five samples of the NIPS DNA sequencing library. So, a piece of E-Gels can run thirty samples. Per the manufacturer’s instructions: 100 ng DNA per well was loaded on E-Gel EX Gels, 2% (Invitrogen, Carlsbad, CA, USA), a pre-cast 2% agarose gel with 0.8% SYBR stain; the gel was run on E-Gel iBase Power System (Invitrogen, Carlsbad, CA, USA) for approximately 15 min; and DNA with target sizes was retrieved from the bottom wells on the gel. A 50 bp DNA ladder was used as the marker (Invitrogen). The selected library was then tested with an Agilent 2100 Bioanalyzer (Fig. 5) and quantified by real-time polymerase chain reaction (PCR) using KAPA Library Quantification Kits (for the Ion Torrent platform).

### Sequencing and data analysis

The selected libraries were pooled together with different barcodes and sequenced using an Ion Proton system (Life Technologies). All sequencing data were aligned to the human genome reference sequences (version: NCBI Build37/hg19) using TMAP software (version 4.6.11). Duplicate reads were removed using the Picard software. Unique reads whose mapping quality score (MAPQs) were greater than 10 and whose lengths were longer than 35 bp were used in subsequent analyses^25^.

In the NGS data analysis for each sample, all referenced chromosomes were divided into segments of the same size (20 kb) bins. The number of unique reads and GC content (rounded to 0.1%) in each 20-kb bin were determined. We then filtered bins without any reads or bins with ‘N’ in the sequences. The remaining bins were corrected and normalized based on their GC content by the LOESS regression^6^. Finally, the percentage of each chromosome was calculated by the total uniquely mapped reads on the target chromosomes divided by the total uniquely mapped reads on all autosomes^7^. To identify abnormal chromosomes for each sample, the z-score for the target chromosome of interest (chr13/18/21) was evaluated with the U-tests method^6^. For classification of the target chromosome’s aneuploidy state, we used a z-score of more than 4.00 to classify the chromosome as affected and a z-score of less than 2.58 to classify a chromosome as unaffected. Samples that had a z-score between 2.58 (confidence level = 99%) and 4.00 (confidence level = 99.99%) were classified as “no call”^26^.

### Estimation of fetal DNA fraction

The proportion of reads from chromosome Y (%chrY) was also calculated and was used to determine the fetal DNA fraction in maternal plasma samples collected from pregnancies with male foetuses. The formula we used to calculate fetal DNA fraction was reported by Chiu *et al*.^22^:

$${\rm{Fetal}}\,{\rm{DNA}}\,{\rm{fraction}}\,({\rm{FF}})=\frac{ \% chr{Y}_{MF}- \% chr{Y}_{FF}}{ \% chr{Y}_{AM}- \% chr{Y}_{FF}}$$

where *%chrY*_*MF*_ is the Y chromosomeg a male fetus, *%chrY*_*FF*_ is the background average Y chromosomal percentage of all the women with euploid female fetuses (containing 100% female DNA), and *%chrY*_*AM*_ is the average Y chromosomal fraction among cfDNA in the plasma of three adult men (0.170%).

To determine fetal DNA fraction pregnancies with female fetuses, we applied SeqFF^27^, which was a robust method based on a multivariate model for estimating fetal DNA fraction in pregnant women plasma (Supplemental Fig. 1).

### Validation of different fetal DNA fractions for iNIPS

We collected a blood sample from a pregnant woman carrying a single male fetus with trisomy 21 whose karyotype was confirmed by high-throughput sequencing and chromosome karyotype analysis. The fetal DNA fraction in her plasma was 19.5%. To prepare a fetal DNA fraction gradient, we recruited 30 non-pregnant women, extracted cell-free plasma from their whole blood samples, and constructed sequencing libraries. We then established samples with six fetal DNA fractions (1%, 2%, 3%, 4%, 5% and 10%) by mixing the libraries from the pregnant woman and the 30 non-pregnant women. Thirty samples were prepared for each fraction, and all samples were analysed by NIPS and iNIPS separately to determine the sensitivities of the two methods.

## Electronic supplementary material

Supplementary Information

## Data Availability

The data mentioned in the manuscript are available from the corresponding authors on reasonable request.
