# Supplementary material for: Enrichment of the fetal fraction in non-invasive prenatal screening reduces maternal background interference
Source: Sci Rep. 2018 Dec 5;8:17675. doi: 10.1038/s41598-018-35738-0 (PMC6281613; doi:10.1038/s41598-018-35738-0)
Supplement: Supplementary file 1 — Supplementary Information [file 41598_2018_35738_MOESM1_ESM.pdf]

Supplementary Information for

Enrichment of fetal fraction for non-invasive prenatal screening reduces maternal background interference

Bo Liang[1], Hong Li[2], Quanze He[2], Haibo Li[2], Lingyin Kong[1], Liming Xuan[1], Yingying Xia[1], Jingjing Shen[1], Yan Mao[1], Yixue Li[1], Ting Wang[2*], and Yi-Lei Zhao[1*]

[1]State Key Laboratory of Microbial Metabolism, Joint International Research Laboratory of Metabolic and Developmental Sciences, School of Life Sciences and Biotechnology, Shanghai Jiao Tong University, 800 Dongchuan Road, Shanghai 200240, China
[2]Center for Reproduction and Genetics, The Affiliated Suzhou Hospital of Nanjing Medical University, 38 Fengjin Road, Wuzhong, Suzhou, Jiangsu 215002, China

[*]Corresponding authors: yileizhao@sjtu.edu.cn (Y.Z); biowt@163.com (T.W)

Supplemental Table 1. Fetal fraction of 50 samples by NIPS and iNIPS.

Sample ID	Fetal fraction (%) by NIPS	Fetal fraction (%) by iNIPS	Increase multiple	Sample ID	Fetal fraction (%) by NIPS	Fetal fraction (%) by iNIPS	Increase multiple
S1	22.83%	39.75%	1.74	S26	8.02%	14.70%	1.83
S2	8.31%	15.34%	1.85	S27	19.43%	43.68%	2.25
S3	13.22%	25.18%	1.9	S28	18.25%	38.58%	2.11
S4	15.35%	27.35%	1.78	S29	10.08%	33.66%	3.34
S5	20.66%	35.38%	1.71	S30	5.73%	20.04%	3.5
S6	12.16%	39.17%	3.22	S31	11.60%	28.72%	2.48
S7	14.75%	34.78%	2.36	S32	7.75%	22.57%	2.91
S8	15.77%	39.20%	2.49	S33	9.57%	28.48%	2.98
S9	5.19%	14.27%	2.75	S34	8.29%	29.47%	3.55
S10	10.64%	26.47%	2.49	S35	9.86%	36.50%	3.7
S11	10.74%	30.90%	2.88	S36	27.97%	65.67%	2.35
S12	9.35%	26.23%	2.81	S37	7.79%	21.61%	2.77
S13	6.57%	21.53%	3.28	S38	7.24%	20.35%	2.81
S14	8.94%	25.69%	2.87	S39	18.32%	49.30%	2.69
S15	5.91%	9.63%	1.63	S40	12.92%	35.28%	2.73
S16	7.54%	15.16%	2.01	S41	17.64%	39.87%	2.26
S17	6.15%	12.47%	2.03	S42	3.87%	12.36%	3.19
S18	21.37%	47.73%	2.23	S43	5.60%	16.32%	2.91
S19	7.57%	20.41%	2.7	S44	8.53%	23.73%	2.78
S20	11.34%	26.38%	2.33	S45	14.47%	42.81%	2.96
S21	15.47%	39.40%	2.55	S46	4.26%	10.58%	2.48
S22	13.06%	33.98%	2.6	S47	4.96%	13.26%	2.67
S23	9.92%	19.89%	2.01	S48	6.62%	18.81%	2.84
S24	12.74%	21.36%	1.68	S49	7.90%	19.07%	2.41
S25	14.21%	26.55%	1.87	S50	12.24%	29.47%	2.41

Supplemental Table 2. Chromosome abnormality derived from maternal DNA.

Sample ID	Karyotype of mother	Z-score (NIPS)	Abnormality Fraction (NIPS)	Z-score (iNIPS)	Abnormality Fraction (iNIPS)	Decreased Fraction	Decreased level
221103031513	45,X[12]/46,XX[38]	-25.13	33.5%	-15.40	20.5%	13.0%	1.6
221103031512	47,XXX	40.64	93.4%	31.27	78.4%	15.0%	1.2

Sample ID: the identification of the two plasma samples.
Karyotype of mother: the karyotype of the two pregnant women.
Z-score (NIPS): the z-score detected by NIPS.
Abnormality Fraction (NIPS): the fraction of abnormal chromosomes detected in the plasma by NIPS. Since the abnormality was derived from the mother, it was a false positive for fetal chromosome abnormality detection.
Z-score (iNIPS): the z-score detected by iNIPS.
Abnormality Fraction (iNIPS): the fraction of abnormal chromosomes detected in the plasma by iNIPS.
Decreased Fraction: the abnormal fraction decreased by iNIPS comparing with NIPS.
Decreased level: the times decreased by iNIPS comparing with NIPS.

Supplemental Table 3. Thresholds of sizes of maternal chromosome abnormalities that can cause false positives if fetal fraction is 4%.

Chromosome	Effective length(Mb)#	Threshold (Mb)*	Chromosome	Effective length(Mb) #	Threshold (Mb)*
chr1	228	9.50	chr13	96	4.00
chr2	237	9.88	chr14	88	3.67
chr3	188	7.83	chr15	80	3.33
chr4	187	7.79	chr16	78	3.25
chr5	176	7.33	chr17	78	3.25
chr6	166	6.92	chr18	74	3.08
chr7	155	6.46	chr19	47	1.96
chr8	141	5.88	chr20	59	2.46
chr9	123	5.13	chr21	34	1.42
chr10	131	5.46	chr22	33	1.38
chr11	131	5.46	chrX	150	6.25
chr12	129	5.38			

#Effective lengths of chromosomes are based on GRCh37.p13 (https://www.ncbi.nlm.nih.gov/grc/human/data?asm=GRCh37.p13) released by the Human Genome Project (HGP), without telomeres, centromeres, satellite, and other heterochromatic regions.

*The thresholds of the sizes of maternal chromosome abnormalities that can cause false positives are based on fetal fraction and following is the formula for calculating the thresholds:

$$\text{Threshold} = \text{Effective length} \times \text{fetal fraction}/\text{maternal fraction} = \text{Effective length} \times \text{fetal fraction}/(1 - \text{fetal fraction})$$

For example, if there was a maternal microduplication of 10Mb on chromosome X and the raw fetal DNA fraction was 4%, the threshold would be 6.25Mb (150Mb×4%/96%), i.e. maternal copy number variation lager than 6.25Mb would cause a false positive in fetal abnormality detection by NIPS; applied to iNIPS, the fetal DNA fraction would increase to 10%, and then the threshold would also increase to 16.67Mb (150Mb×10%/90%), so it would not interfere with iNIPS.

Supplemental Table 4. Fetal fraction of 22 “no call” samples by NIPS and iNIPS.

Sample No.	NIPS		iNIPS	
	%SeqFF	%ChrY*	%SeqFF	%ChrY*
1	9.30%	7.01%	27.23%	21.65%
2	8.19%	NULL	25.17%	NULL
3	13.22%	NULL	29.16%	NULL
4	6.63%	6.39%	14.76%	17.72%
5	12.04%	NULL	31.91%	NULL
6	7.13%	5.35%	27.22%	23.16%
7	8.70%	NULL	18.58%	NULL
8	5.91%	NULL	13.02%	NULL
9	9.97%	7.38%	20.35%	17.43%
10	10.72%	13.10%	37.53%	41.84%
11	5.43%	NULL	9.32%	NULL
12	11.03%	14.05%	31.19%	28.68%
13	10.26%	9.15%	17.68%	22.02%
14	6.62%	7.96%	15.97%	20.79%
15	5.91%	NULL	18.58%	NULL
16	6.02%	9.28%	21.86%	26.79%
17	9.01%	8.26%	18.58%	12.51%
18	11.53%	NULL	21.86%	NULL
19	7.47%	4.28%	8.94%	5.92%
20	15.74%	16.65%	39.79%	44.42%
21	6.39%	NULL	14.75%	NULL
22	4.37%	4.47%	9.39%	11.78%

*The fetal fraction of pregnant women with pregnancies with female fetuses by %ChrY was described as “NULL”.

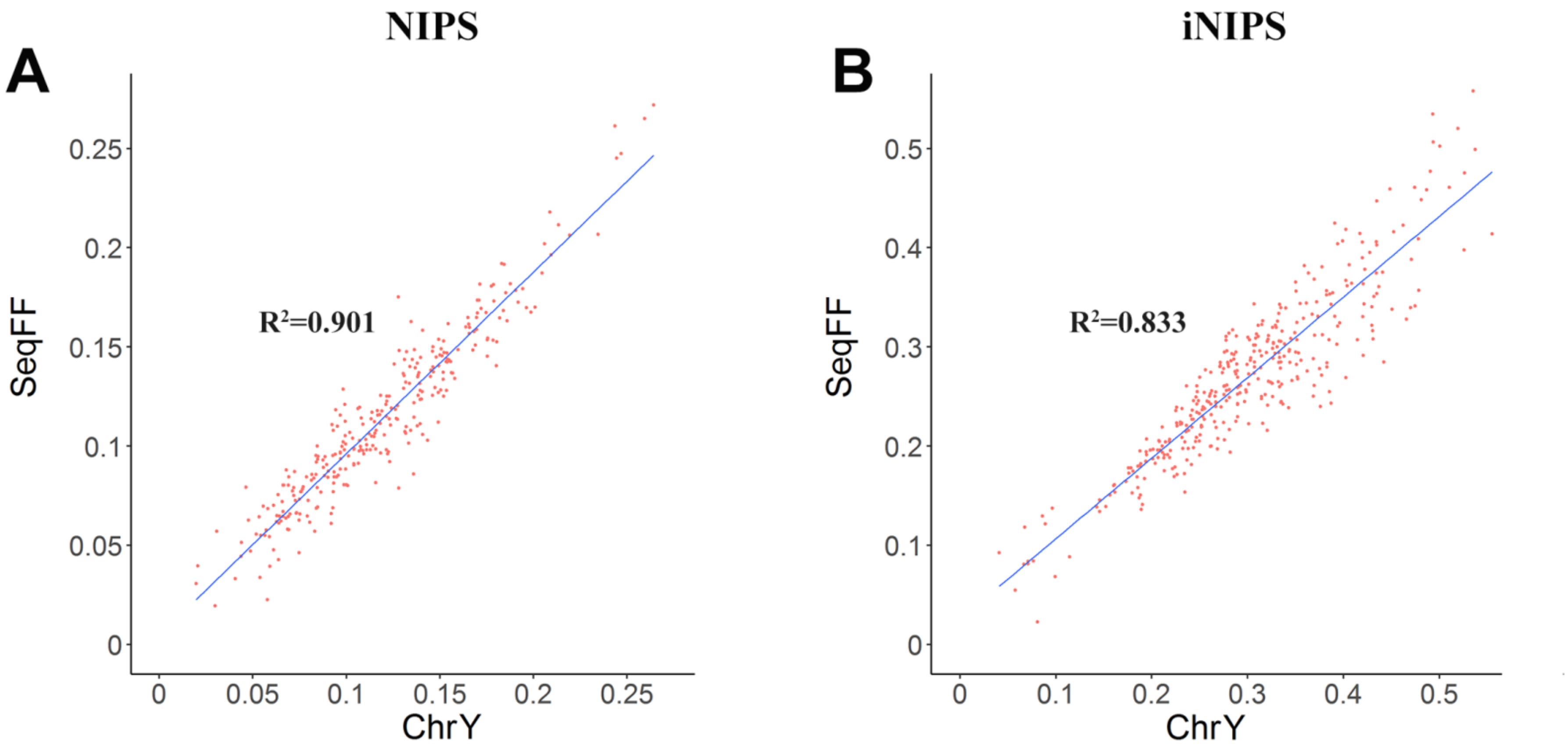

Supplemental Figure 1. The comparison of fetal fraction estimated by SeqFF and %ChrY. (**A**) The fetal fraction in NIPS estimated by SeqFF and %ChrY. (**B**) The fetal fraction in iNIPS estimated by SeqFF and %ChrY.

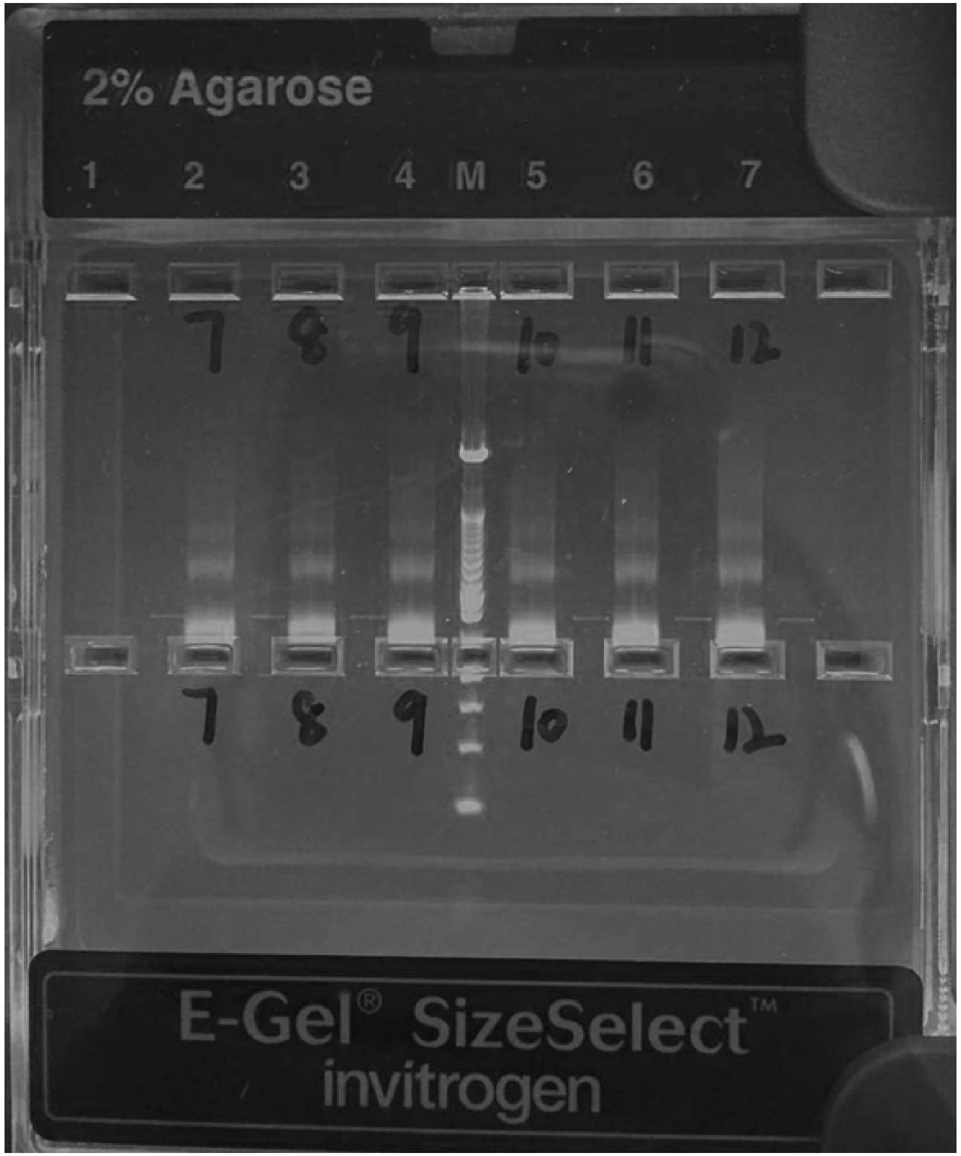

Supplemental Figure 2. The full-length E-gel for library size selection. Lane 1 and Lane 8 were empty lanes, Lane 2–4 contained iNIPS samples, Lane M contained markers, and Lane 5–7 were samples of other study.
